# Understanding of BeCu Interaction Characteristics with a Variation of ns Laser-Pulse Duration

**DOI:** 10.3390/ma11081423

**Published:** 2018-08-13

**Authors:** Dongkyoung Lee

**Affiliations:** Department of Mechanical and Automotive Engineering, Kongju National University, Cheonan 31080, Korea; ldkkinka@kongju.ac.kr

**Keywords:** laser-material interaction characteristics, laser spot cutting, spring contact probe, semiconductor package inspection, beryllium copper

## Abstract

An inspection process using a Spring Contact Probe (SCP) is an essential step in the semiconductor-manufacturing process. Many plungers, which are the main body of the SCP, are manufactured by a stamping process. After the stamping process, mechanical cutting is applied and the plunger body may be damaged. Thus, to improve cut quality and productivity while minimizing body damage, laser spot cutting can be used. To fully utilize this technology, it is necessary to investigate interaction characteristics of beryllium copper (BeCu) during laser spot cutting. Effects of a total irradiated laser-pulse energy (1 mJ~1000 mJ) and pulse duration (100 ns~8 ns) on the material-removal zone, thermal depth, and crater size are examined. The crater size can be affected by the localization of heating dominantly. An incubation model is applied to investigate the correlation between crater size and laser-pulse energy. Surface morphology characteristics such as edge separation, small particles, spatter motion, and soaring-up motion are observed.

## 1. Introduction

After semiconductor chips are fabricated on wafers, they are divided into individual semiconductor chips. After packaging these semiconductor chips, to be protected from mechanical stress, the packaged semiconductor devices have to be electrically tested. The semiconductor devices with manufacturing defects are classified during the electronic test. Many spring-loaded contact probes are used to test electrical connectivity between a Printed Circuit Board (PCB) and the semiconductor devices [1]. These probes are called Spring Contact Probes (SCP) [2]. A typical composition of an SCP is a plunger, a barrel, and an internal spring [3], as shown in Reference [4].

A major material of plungers is beryllium copper (BeCu). The plungers are typically coated with gold and this coating improves electrical performance and corrosion resistance. BeCu is a copper alloy with 0.5~3% beryllium and it has high strength, high conductivity, is nonmagnetic, nonsparking, etc. Thus, BeCu can be widely used in metalworking and has many specialized applications, such as musical instruments, precision-measurement devices, electrical connectors, automotive systems, and aerospace systems. The plunger tip is to make contact with the PCB and semiconductor package. A stamping process is applied to manufacture plungers [5] so that a number of plungers are molded together to improve productivity, as shown in Reference [4]. After the stamping process, plunger connection arms need to be cut into pieces. A current cutting method is mechanical cutting. Mechanical cutting may damage the body of plunger owing to the mechanical force applied at the cutting interface [6]. Furthermore, connection arms may be deflected so that fractures can eventually occur because of mechanical-stress accumulation. Moreover, cutting tools wear over time and this tool wear results in process instability and poor cut quality. Poor cut quality may affect electric connectivity between SCP and semiconductor so that electrical performance could be measured incorrectly. In that case, inspection performance and a production yield would be decreased. Therefore, these problems can be solved by applying laser spot cutting. In addition, laser spot cutting may also improve both cut quality and productivity.

Laser cutting is a popular application among laser-aided manufacturing [4,7,8] since it has various advantages, such as being contact-free process, having high-energy concentration, fast processing time, a small Heat-Affected Zone (HAZ), and applicability to almost every material [9]. Thus, laser cutting has been applied to many different types of materials, such as sandwiched composites [10,11,12,13,14,15,16,17,18,19,20,21,22,23], reinforced composites [9,24,25,26,27,28,29], and metals [4,6,8,13,30,31,32,33,34,35,36]. Laser spot cutting is using a laser to separate a workpiece into two or more pieces [4]. Since there is no relative motion, this may make the cutting process more efficient. To examine optimal laser parameters, interaction characteristics between BeCu and laser need to be fully understood. However, there is little information about the interaction characteristics. Therefore, this study examines crater size, thermal depth, and ablation threshold to evaluate ablation characteristics. The ablation threshold and incubation coefficient are compared with the literature. In addition, ablation depth, full penetration, and material-removal zone are evaluated. This paper is composed as follows: First, a sample and experimental setup are described. Second, interaction characteristics are evaluated and discussed. Finally, conclusions are summarized.

## 2. Experiments

**Figure 1:**
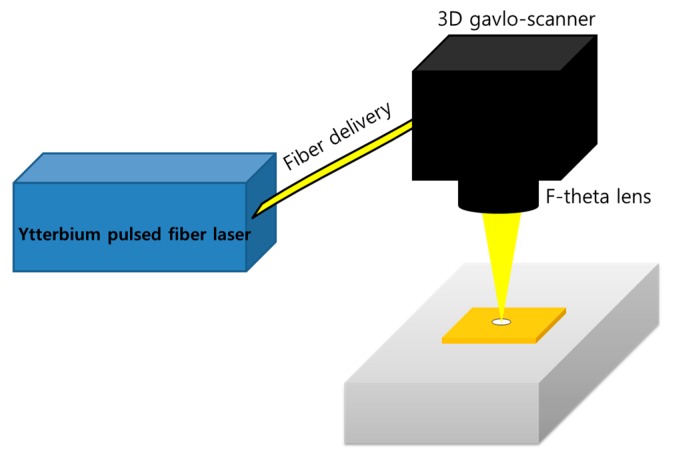
Schematic of experimental setup.

47-µm-thick BeCu, which is a copper alloy with 1.8~2% beryllium, was prepared using the stamping process. On the top and bottom of the BeCu, 4-µm-thick Au was coated by electroplating so that the total thickness was 55 µm. Rectangular samples shown in Reference [4] were used. Due to the low investment cost, a nanosecond laser or Ytterbium pulsed-fiber laser (IPG-YLPM, IPG photonics, Oxford, MA, USA) was chosen. The experimental setup is shown in Figure 1. The laser-pulse durations were controllable in the range of 4 ns to 200 ns. Pulse durations chosen for the experiments were 8, 20, 50, and 100 ns. Chosen laser-pulse durations provided enough time for thermal-energy propagation [37]. This created a relatively large melt pool and evaporation could also occur. Wavelength was 1064 nm. The maximum average output laser power was 20 W and the maximum average power was fixed for experiments to obtain high productivity. The Gaussian laser beam was focused on the top surface and spot size was 30 µm at a focal position. A 3D galvoscanner (RAYLASE AS-12Y, Raylase, Wessling, Germany) was used to deflect the laser beam from the laser source to the workpiece. Assistant or shielding gas was not used.

**Figure 2:**
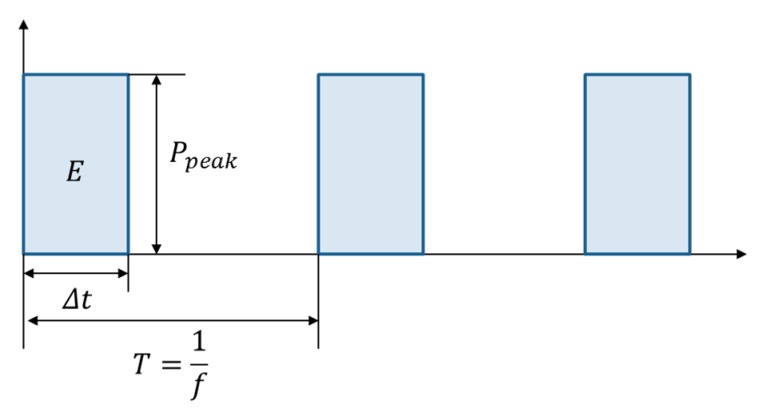
The relationship between pulse energy and laser power.

Two variables, which are pulse duration and total irradiated laser energy, were chosen for independent variables. The repetition rate was modified to maintain the maximum average-output laser power for all laser-pulse durations (100 ns~8 ns). For example, when the repetition rate was set to 40 kHz for 100 ns pulse duration, the multiplication of the repetition rate (40 kHz) and pulse energy (500 µJ) led to the average power of 20W. In addition, when the repetition rate was set to 200 kHz for 8 ns pulse duration, the multiplication of the repetition rate (200 kHz) and pulse energy (100 µJ) also led to the average power of 20 W. Laser parameters used were tabulated in Table 1. Average output laser power was set to 20 W. A pulse energy (E) and total irradiated laser energy ($E_{total}$) can be calculated as

(1) $$\;E_{total}=E\cdot N=\left(P_{peak}\Delta t\right)\cdot N=\left(\frac{P_{avg}}{f}\right)\cdot N\;\;$$

where E is the pulse energy, $P_{avg}$ is the average output laser power, Δt is the pulse duration, $P_{peak}$ is the peak pulse power, N is the number of pulses, and f is the repetition rate. This relationship is shown in Figure 2. Since $P_{avg}$ was set to the maximum power, or $P_{avg}=20\;W$, the pulse energy was inversely proportional to f. The pulse energy for each pulse duration is shown in Table 1. To set the same $E_{total}$, N was adjusted for each pulse duration. N used for experiments is shown in Table 1. The laser pulses were applied to the same spot at the constant fluence. A confocal microscope (OLS4000, Olympus, Tokyo, Japan) was utilized to measure the ablation profile. In addition, a Scanning Electron Microscope (SEM) (Tescan-Vega3, Zeiss, Oberkochen, Germany) was used to observe the interaction characteristics.

## 3. Results and Discussions

Ablation characteristics, depending on total irradiated laser energy ($E_{total}$) and pulse duration, were observed. Crater size, ablation threshold, thermal depth, and incubation coefficient were observed. The crater size was measured based on the maximum distance of the melting zone. Thermal depth could determine the localization of the heating,

(2) $$\;l_{th}=\sqrt{\alpha \Delta t}\;$$

where $l_{th}$ is the thermal depth, and α and Δt are the thermal diffusivity and pulse duration, respectively. Material-removal zone and ablation depth were investigated. The interaction characteristics and surface morphology were discussed in detail. In this paper, crater size and material-removal zones were measured from the top surface in length scale due to radial symmetricity.

### 3.1. Crater Size, Thermal Depth, Ablation Threshold, and Incubation Coefficient

**Figure 3:**
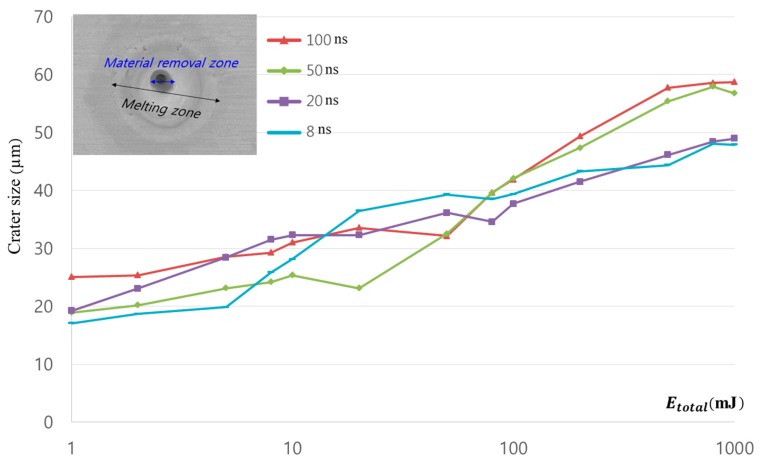
Comparison of crater size.

Measured craters are shown in Figure 3. The *x*-axis of Figure 3 is total irradiated laser energy ($E_{total}$) and plotted in a log scale. As $E_{total}$ increased, the crater size increased logarithmically in all pulse durations. At $E_{total}=\;$1 mJ, the total number of pulses was less than 10. Hence, less number of pulses led to localized heat. In addition, heat conduction rather than evaporation was predominant since electron temperature was equilibrated with the atoms at the nanosecond laser-pulse duration. This caused strong heating of the irradiated volume [38]. Because majority part of the sample was Cu, the material properties of Cu were used. The α of Cu was 1.15 ${\mathrm{cm}}^{2}/s$. The thermal depth for all pulse durations was tabulated in Table 2.

Even though thermal depth monotonically increased from 0.959 to 3.391 μm as pulse duration increased, the variation of crater size was insignificant. This may have been related to the direction of heat transfer. Since thermal depth was the indirect indicator of heat effect in longitudinal direction and the crater size was highly affected by the heat transfer in transverse direction, correlation between thermal depth and crater size was insignificant.

**Figure 4:**
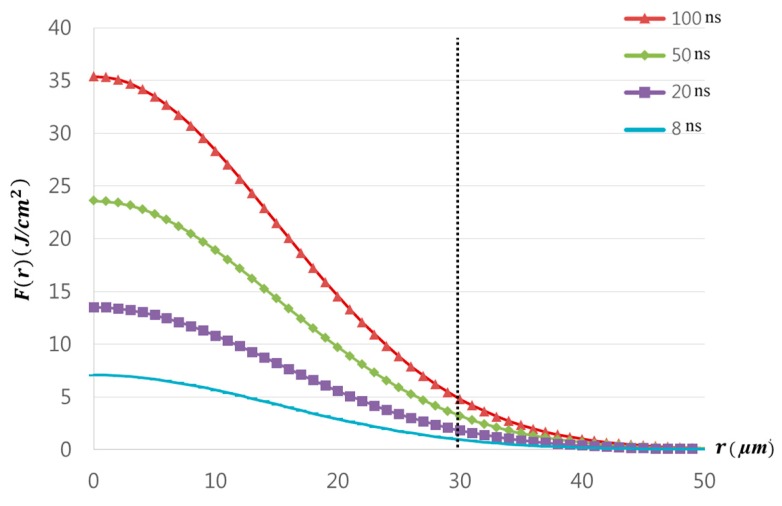
A Gaussian spatial distribution of the laser beam.

Since the 8 ns pulse duration had the minimum thermal depth among the other pulse durations, a much-localized heat effect could have been expected. Therefore, the minimum crater size was observed in the low $E_{total}$, which was more sensitive to localized heat. The crater size increased sharply for the pulse duration of 50 and 100 ns when $E_{total}$ was over 100 mJ. For 20 ns pulse durations, crater size decreased slightly, at which $E_{total}$ was 80 mJ. The decreasing crater size proved physically meaningful due to surface-morphology variation. This morphology change will be discussed in detail in the next section. When $E_{total}$ was high, the differences in crater size between the pulse durations of 50 and 100 ns, as well as between the pulse durations of 8 and 20 ns, were hardly observed. While a small amount of $E_{total}$ led to the localization of heating, three kinds of thermal processes, i.e., vaporization, normal boiling, and explosive boiling, were influencing the laser–material interaction as increasing energy [39,40,41,42,43]. Crater sizes less than a laser-spot size, or 30 μm, were observed at the point where low $E_{total}$ was applied. To explain this, an ablation threshold and a Gaussian laser-beam distribution needed to be introduced first. The ablation threshold can be expressed in terms of peak fluence. The peak fluence of a Gaussian beam can be calculated by

(3) $$\;F_{o}=\frac{2E}{\pi w_{0}^{2}}\;$$

where E is a pulse energy and $w_{0}$ is a Gaussian beam radius. According to Equation (4), the calculated laser fluences are 7.07 $J/{\mathrm{cm}}^{2}$, 13.5 $J/{\mathrm{cm}}^{2}$, 23.6 $J/{\mathrm{cm}}^{2}$, and 35.4 $J/{\mathrm{cm}}^{2}$ for the pulse duration of 8 ns, 20 ns, 50 ns, and 100 ns, respectively. Since the ablation threshold of Cu with a nanosecond laser pulse was given in the range of 5.1 $J/{\mathrm{cm}}^{2}$ to 11 $J/{\mathrm{cm}}^{2}$ [38,44,45], the fluence used for all cases except the 8 ns pulse duration was greater than the ablation threshold of Cu. Thus, having a crater size less than 30 μm was understandable for the 8 ns pulse duration. However, other cases also showed crater size less than 30 μm. This may be explained by examining laser-beam distribution. When a laser beam has the Gaussian distribution, the fluence also has the Gaussian distribution. The spatial fluence distribution for the Gaussian laser beam is given by Reference [39]:

(4) $$\;F\left(r\right)=F_{0}e^{\frac{-2r^{2}}{w_{o}^{2}}}\;$$

where $F_{0}$ is peak fluence in the beam and r is radius. According to the fluence distribution, the fluence near the edge of the laser-beam spot was less than 10 $J/{\mathrm{cm}}^{2}$, which is the ablation threshold of Cu. This is shown in Figure 4. Therefore, crater size less than 30 μm could be observed when $E_{total}$ was less than 10 mJ in all cases.

The peak fluence ($F_{0})$ can be calculated with the relationship between an ablation threshold fluence $\left(F_{th}\right)$ and the diameter (*D*) [46]:

(5) $$\;D^{2}=2w_{0}^{2}\ln\left(\frac{F_{0}}{F_{th}}\right)\;$$

Furthermore, the incubation model explains accumulation behavior [47]. This model explains the relationship between the singe-shot ablation threshold fluence and the ablation threshold fluence for the number of laser pulses (*N*). This relationship can be expressed as follows:

(6) $$\;F_{th}\left(N\right)=F_{th}\left(1\right)N^{s-1}\;$$

where $F_{th}\left(1\right)$ is the ablation threshold fluence using one laser pulse and *s* is the incubation coefficient. The crater size and the number of laser pulses are related by combining Equations (6) and (7):

(7) $$\;D=w_{0}\sqrt{2\ln\left(\frac{F_{0}}{F_{th}\left(1\right)N^{s-1}}\right)}\;$$

where $F_{0}$ is the ablation fluence [39]. Equation (8) can be rearranged as:

(8) $$\;-\frac{1}{2}{\left(\frac{D}{w_{0}}\right)}^{2}+\ln F_{0}=\ln\left(\;F_{th}\left(1\right)\right)+\left(s-1\right)\ln\left(N\right)\;$$

**Figure 5:**
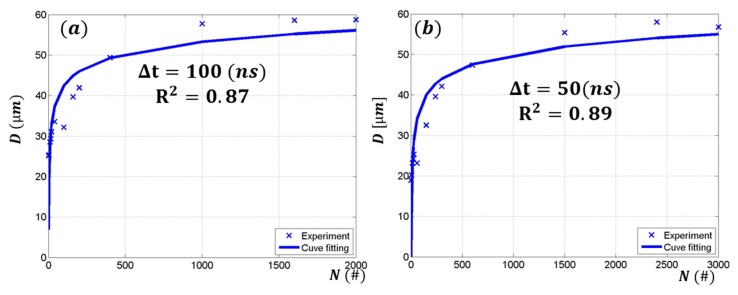

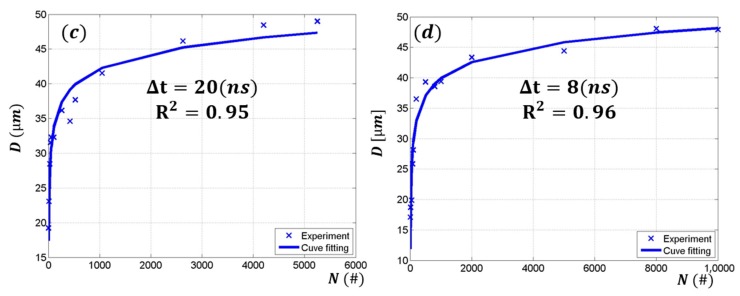
Crater diameter VS the number of laser pulses applied to the same spot at the constant fluence for the (**a**) 100 ns; (**b**) 50 ns; (**c**) 20 ns; and (**d**) 8 ns laser-pulse duration.

From Equation (6), $F_{th}\left(1\right)$ and s can be obtained by linear interpolation and substitution. The single-shot ablation threshold fluence and incubation coefficient are tabulated in Table 2. Figure 5 shows the crater diameters versus the number of laser pulses applied to the same spot at a constant fluence. The solid line shows a curve fitting according to Equation (8). The single-shot ablation threshold fluences are 40.9 $J/{\mathrm{cm}}^{2}$, 36.3 $J/{\mathrm{cm}}^{2}$, 14.8 $J/{\mathrm{cm}}^{2}$, and 9.79 $J/{\mathrm{cm}}^{2}$ for the 100 ns, 50 ns, 20 ns, and 8 ns pulse duration, respectively. The incubation coefficients were 0.75, 0.74, 0.84, and 0.82 for the 100 ns, 50 ns, 20 ns, and 8 ns pulse duration, respectively. Attained ablation threshold for the 8 ns pulse duration is very similar to the literature, where the ablation threshold is observed in the range of 5.1 $J/{\mathrm{cm}}^{2}$ and 11 $J/{\mathrm{cm}}^{2}$ [38,44,45]. However, ablation thresholds for the other pulse durations are hardly found from the literature. The coefficient of determination ($R^{2}$) is shown for each pulse duration in Figure 5. The short pulse duration (8 and 20 ns) resulted in a relatively good fitting and high $R^{2}$ values. However, the long pulse duration (50 and 100 ns) showed low $R^{2}$ values. Furthermore, $R^{2}$ value decreased as the pulse duration increased. This may have been due to heat accumulation effect. Since longer pulse duration leads to deeper thermal depth and longer exposure time to laser energy, heat accumulation may have been more pronounced. Thus, wider and deeper melting-zone formation may result in the deviation.

### 3.2. Material-Removal Zone, Ablation Depth, and Full Penetration

**Figure 6:**
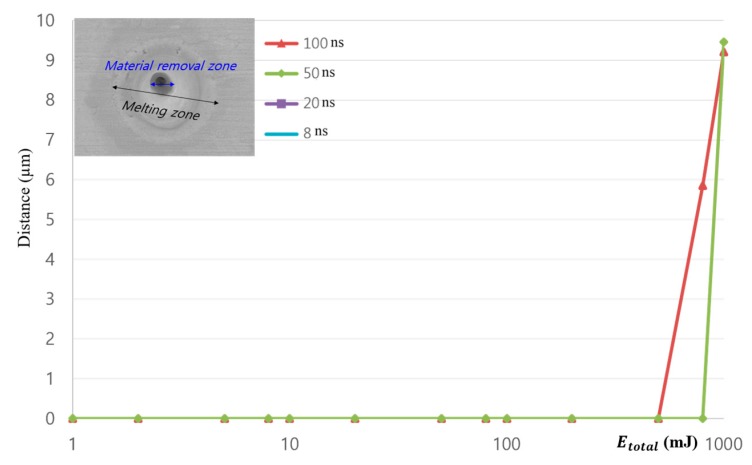
Comparison of material-removal zones.

The material-removal zone is plotted in Figure 6. The *x*-axis of Figure 6 is plotted on a log scale. At the 100 ns and 50 ns pulse duration, material-removal zones were detected when $E_{total}$ was greater than 500 mJ and 800 mJ, respectively. All the material-removal zones were less than 10 μm. No material = removal zone was found when the pulse duration was less than or equal to 20 ns. From the figure, the given $E_{total}$ is the proper range to investigate the ablation regime since the given $E_{total}$ was not enough to remove the material.

**Figure 7:**
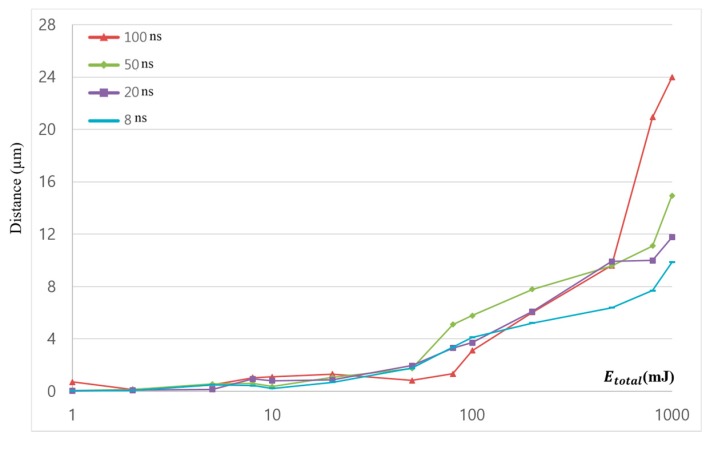
Comparison of ablation depth.

Ablation depth was measured by a confocal microscope. Comparison of ablation depth is shown in Figure 7. The *x*-axis of Figure 7 is total irradiated laser energy ($E_{total}$) and plotted in a log scale. Higher peak power caused more ablation if we consider only one laser-pulse duration. However, this understanding was not applicable in this study. This is because the independent variable was not the number of laser pulses using one type of laser-pulse duration, but the total irradiated laser energy. Furthermore, one laser pulse was not enough to clearly ablate the material for the given material. In addition, the minimum $E_{total}$, or 1 mJ, required a different number of laser pulses, depending on the pulse duration. Therefore, the common understanding that higher peak power leads to more ablation is hardly applicable to explain the phenomena.

Ablation depth barely increased when $E_{total}$ was less than 80 mJ. When $E_{total}$ was 80 mJ, the ablation depth suddenly increased. No full penetration was observed in the 100 ns, 50 ns, 20 ns, and 8 ns pulse durations. However, full penetration may be expected as $E_{total}$ increases. Although the same $E_{total}$ was applied for all pulse durations, the ablation depth was different. Apparent differences can be observed at $E_{total}$ = 1000 mJ. According to the laser parameters used in this study, when a longer pulse duration was used, weaker peak power formed, as shown in Table 1. However, a longer pulse duration had higher pulse energy. Thus, ablation depth was highly dependent on $E_{total}$ when $E_{total}$ was greater than 500 mJ.

### 3.3. Effect of Laser Parameters on Ablation Characteristics and Surface Morphology

**Figure 8:**
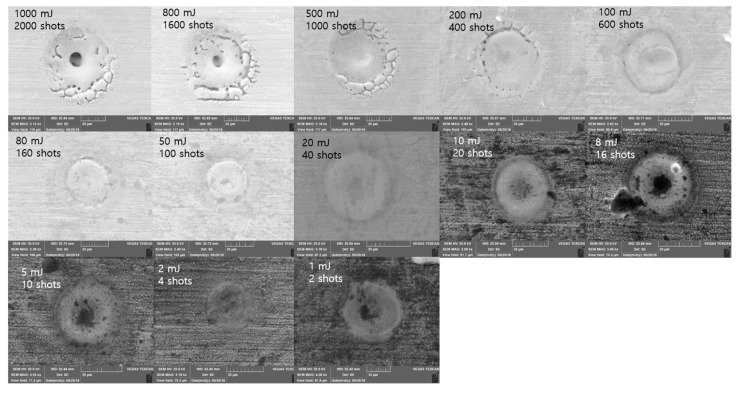
Scanning electron microscope (SEM) images Δ*t* = 100 ns.

Figure 8 shows SEM images for the 100 ns pulse duration. Crater size was clearly observed if $E_{total}$ was less than 80 mJ. These craters were formed by a molten workpiece when there was enough time for the thermal wave to propagate into the target. Thus, this thermal wave created a molten layer and the resolidified molten layer formed the crater [37]. When $E_{total}$ was 100 mJ, the groove was discovered at the edge of the crater. This groove became separated when $E_{total}$ increased into 200 mJ. This edge separation was readily observable if $E_{total}$ was greater than 200 mJ. After the edge separation was detected, the crater size converged into the value of 60 μm. After the edge-separation and crater-size convergence, a material-removal zone was observed. Thus, evaporation was introduced as a material-removal mechanism in addition to the melting and resolidification.

**Figure 9:**
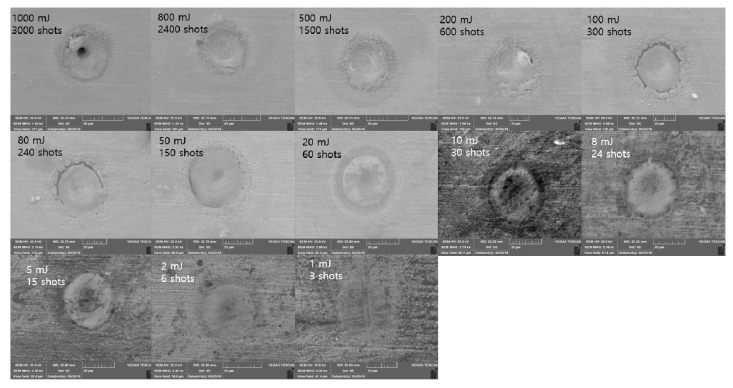
SEM images Δ*t* = 50 ns.

SEM images for the 50 ns pulse duration are shown in Figure 9. In this pulse duration, crater size changed considerably in the range of 18.9 μm to 58.2 μm. The crater existed where $E_{total}$ was less than 10 mJ. When $E_{total}$ was 20 mJ, an initial stage of edge separation was observed around the edge of the crater. Not only edge separation, but also small particles could be observed around the crater edge as increasing $E_{total}$. The particle size was in the range of 300 nm to 1.5 μm. Due to the repeated laser pulses, a molten workpiece was spattered from the laser–material interaction zone to the edge. After the laser–material interaction, this spattered molten workpiece was solidified and it formed particles. Furthermore, these particles may have been formed by condensation of plasma [48]. However, further investigation is required to clearly justify and quantify the causation of these particles. Further increase of $E_{total}$ led to create the material-removal zone when $E_{total}$ was 1000 mJ.

**Figure 10:**
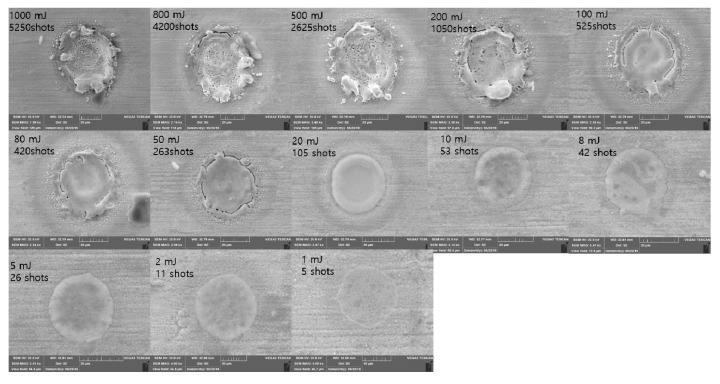
SEM images Δ*t* = 20 ns.

SEM images of the 20 ns pulse duration are shown in Figure 10. Similar phenomena observed from the case of 50 ns pulse duration were observed. Smooth crater surface could be found in the $E_{total}$ range of 1 mJ to 10 mJ. At the $E_{total}$ of 20 mJ, a heat-affected zone was observed around the crater. Both the edge separation and small particles were observed in the $E_{total}$ range of 50 mJ to 100 mJ. When $E_{total}$ increases, the crater showed a soaring-up motion. This soaring-up motion may have been due to the intense evaporation, which gives rise to recoil pressure during the laser–material interaction. Thus, there existed three interaction characteristics, i.e., edge separation, small particles, and soaring-up motion, when $E_{total}$ was greater than 200 mJ.

**Figure 11:**
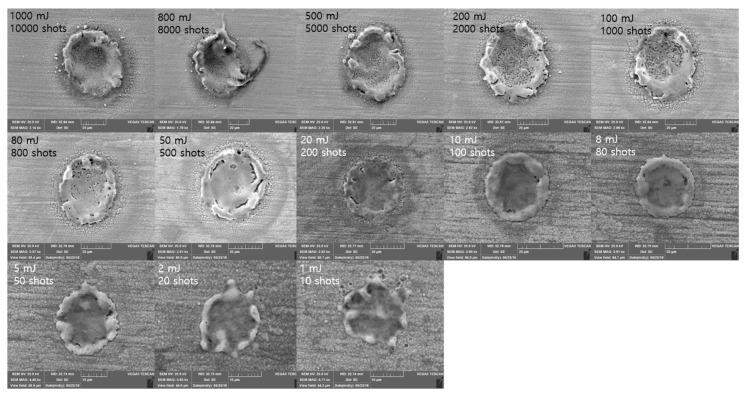
SEM images Δ*t* = 8 ns.

SEM images for the 8 ns pulse duration are shown in Figure 11. Interaction characteristics observed are almost similar to the case for the 20 ns pulse duration. However, the edge separation was rarely observable. The interesting thing was that the soaring-up motion was detected even though $E_{total}$ was less than 10 mJ due to high peak power.

## 4. Conclusions

To fully utilize laser cutting on BeCu, interaction characteristics during laser spot cutting were observed with variation of an ns pulse duration (100 ns~8 ns) and $E_{total}$ (1 mJ~1000 mJ). Crater size, material-removal zone, and thermal depth were examined. Furthermore, ablation depth, ablation threshold, incubation coefficient, and surface morphology were observed. Crater size can be affected dominantly by the localization of heating when a small amount of $E_{total}$ is applied. The relationship between crater size and the number of laser pulses was examined with the incubation model. A single-shot ablation threshold and incubation coefficient obtained from the model were compared. The values are in a good agreement with the literature for the 8 ns laser pulse. Edge separation, small particles, spatter motion, and soaring-up motion were detected on the surface and discussed. The results can be used for BeCu laser cutting in many applications as a fundamental reference.

## Figures and Tables

**Table 1:** Laser parameters used for experiments.

#	Δ*t* (ns)	*f* (kHz)	Pulse *E* (µJ)	*P_peak_* (W)	Total Energy (mJ)												
					1000	800	500	200	100	80	50	20	10	8	5	2	1
					Number of Pulses (#)												
1	100	40	500	5000	2000	1600	1000	400	200	160	100	40	20	16	10	4	2
2	50	60	333.3	6666.7	3000	2400	1500	600	300	240	150	60	30	24	15	6	3
3	20	105	190.5	9523.8	5250	4200	2625	1050	525	420	263	105	53	42	26	11	5
4	8	200	100	125,000	10,000	8000	5000	2000	1000	800	500	200	100	80	50	20	10

**Table 2:** Thermal depth ($l_{th}$), ablation threshold fluence ($F_{th}$) and incubation coefficient (s).

Δt (ns)	$\;l_{th}\;\left(\mu m\right)$	$\;F_{th}\;\left(J/cm^{2}\right)$	s
100	3.391	40.964	0.750
50	2.298	36.317	0.736
20	1.517	14.751	0.844
8	0.959	9.790	0.825
